# A Balancing Act: A Systematic Review and Metasynthesis of Family-Focused Practice in Adult Mental Health Services

**DOI:** 10.1007/s10567-022-00418-z

**Published:** 2022-11-01

**Authors:** M. Tuck, A. Wittkowski, L. Gregg

**Affiliations:** 1 School of Health Sciences, The University of Manchester, Manchester, UK; 2 Greater Manchester Mental Health NHS Foundation Trust, Manchester, UK; 3 Division of Psychology and Mental Health, School of Health Sciences, Faculty of Biology, Medicine and Health, The University of Manchester, Manchester Academic Health Science Centre, Zochonis Building, Brunswick Street, Manchester, M13 9PL UK

**Keywords:** Qualitative synthesis, Mental health, Healthcare professionals, Parental mental illness, Children of parents with mental illness

## Abstract

Parental mental illness is a major international public health concern given its implications for whole families, including children. Family-focused practice (FFP), an approach that emphasises a “whole-family” approach to care, provides an opportunity to mitigate the significant risks associated with parental mental health difficulties. The positive benefits associated with FFP have led to a shift in policy and practice towards prioritising FFP within adult mental health services. However, evidence suggests that FFP remains scarce and is not routine. Research has identified the important role of practitioners in facilitating FFP. The current review identified, synthesised and appraised the international qualitative literature examining adult mental health practitioners’ implementation experiences of FFP. It aimed to provide an evidence-informed account of practitioner experiences of FFP delivery and to identify key recommendations to enhance future FFP outcomes in AMHS. Ovid Medline, PsycInfo, CINAHL plus, EMBASE and Web of Science Core Collection were searched systematically, in line with PRISMA guidance, up to January 2022. The Critical Appraisal Skills Programme (CASP) was used to undertake the quality appraisal prior to a thematic synthesis being conducted. The review was registered on PROSPERO. Nineteen papers, spanning 17 years of research with 469 practitioners, were included. Three main themes and 14 subthemes were developed, representing different aspects of practitioner experiences of FFP delivery. Practitioners’ approach to FFP was variable and influenced by their beliefs about FFP, perceived roles and responsibilities, competence, service setting, and personal parenting status. Practitioners engaged in a balancing act to maintain a dual focus on their service-users and their children, to navigate powerful emotions, and consider multiple perspectives in a biomedical organisational structure that advocates individualised treatment. Although working together unified teams, a greater need for external interagency collaboration was identified. The use of strength-based approaches with clients and dedicated staff resources, within clear guidelines and frameworks, was reported to be necessary to maximise FFP delivery. This review proposes a complex FFP dynamic whereby practitioners engage in a constant balancing act between FFP stakeholders to achieve meaningful FFP outcomes for service-users and their families. Service recommendations are provided.

## Background

Parental mental illness is a major international public health concern given its implications for whole families, including dependent children (Bee et al., 2014; Lagdon et al., 2021; Schrank et al., 2015). Due to a plethora of interrelated factors, the risk of these children developing physical, psychosocial and mental health problems is heightened (Bee et al., 2014; Schrank et al., 2015). Conversely, family dynamics can have reciprocal impacts on parents’ mental health due to the additional burdens and stressors they may face (Barrowclough & Hooley, 2003; Foster et al., 2016). Importantly, adverse outcomes are not an inevitability of parental mental health difficulties (Reupert & Maybery, 2011), particularly if families are offered timely support (Hogg et al., 2013). Thus, the early identification of need and provision of support is a priority to improve the lives of these parents and children (Bee et al., 2014).

Family-focused practice (FFP) emphasises a “whole-family” approach to care (Foster et al., 2013). Foster et al. (2016) identified six core practices of FFP: (1) family care planning and goals setting, (2) family and service liaison, (3) individual and family-focused support, (4) individual and family-focused assessment, (5) psychoeducation and (6) a coordinated system of care between families and services. Nevertheless, five reviews have noted a lack of definitional clarity in relation to FFP (Acri & Hoagwood, 2015; Foster et al., 2016; Gregg et al., 2021; Marston et al., 2016; Smith et al., 2020), particularly within adult mental health services (AMHS) (Foster et al., 2016; Reupert et al.,^ 2018^). FFP offers a means to meet the needs of parents with mental health difficulties and their children (Foster et al., 2016; Reupert et al., 2015) and has been identified to mitigate the risk of adverse outcomes (Foster et al., 2016; Grant et al., 2019; Maybery et al., 2015). These mitigating impacts have led to an international shift towards prioritising FFP within AMHS (Grant et al., 2018; Reupert et al., 2015; Shah-Anwar et al., 2019), which are uniquely positioned to deliver FFP (Eassom et al., 2014; Maybery et al., 2016). In AMHS, family-focused practices include approaches, programmes, interventions, models and frameworks that acknowledge the “whole-family” context of the service-user (Marston et al., 2016; Maybery et al., 2015).

However, there are persistent barriers to FFP delivery and limited evidence of routine implementation of FFP in AMHS (Leenman & Arblaster, 2020; Maybery & Reupert, 2009; Reedtz et al., 2019). Lack of necessary knowledge, skills, confidence as well as a lack of training have been identified as barriers on a practitioner level (Gregg et al., 2021; Shah-Anwar et al., 2019) as well as insufficient organisational policy, management and resources on an organisational level (Grant et al., 2019; Gregg et al., 2021; Maybery & Reupert, 2009). Importantly, practitioner factors such as knowledge, attitudes, parental experiences, perceptions of support and training have been demonstrated to be predictors of FFP delivery (Grant et al., 2019; Gregg et al., 2021; Maybery et al., 2016). Allchin et al. (2021) have developed a model for sustaining FFP in AMHS, which acknowledges the important role of practitioners as well as their interrelationship with service-users, families, organisations and the wider socio-political context of FFP operation.

Given adult mental health practitioners’ important role in implementing FFP, a comprehensive understanding of their experiences is imperative to facilitate practical implementation. Previous reviews focused on the prevalence of parents in psychiatric services (Maybery & Reupert, 2018), defining FFP (Foster et al., 2016), the feasibility and effectiveness of FFP interventions (Acri & Hoagwood, 2015; Bee et al., 2014; Schrank et al., 2015), barriers to FFP delivery (Maybery & Reupert, 2009; Shah-Anwar et al., 2019) and FFP implementation factors (Eassom et al., 2014; Gregg et al., 2021).

Maybery and Reupert’s (2009) review provided an informative overview of the factors that impede FFP within AMHS. A particular strength lay in the authors’ efforts to incorporate client and family factors that influence FFP delivery; however, an increase in FFP research means an update is warranted. Shah-Anwar et al. (2019) review of nine qualitative studies focussed on mental health professionals’ perspectives and experiences of FFP, with an emphasis on perceived barriers across both child and adult settings. Conclusions were similar to those of Maybery and Reupert (2009) and highlighted the importance of the organisational context and policies supportive of FFP and clinicians’ attitudes, knowledge and practice. Shah-Anwar et al.’s (2019) review has been a valuable addition to the literature; however, it did not include a number of seminal papers and additional studies have been conducted since their final search date in March 2018. Notably, the inclusion of child services in the sample hinders a more specific AMHS understanding. Similarly, Gregg et al. (2021) provided a mixed-method review centred on the modifiable factors that influence the FFP of adult mental health practitioners. However, since Gregg et al.’s final search was completed in November 2018, there has been several additional qualitative papers since their searches were conducted.

To date, there has been no qualitative review encompassing practitioners’ implementation experiences of FFP including facilitators as well as barriers, in AMHS alone, which has its own unique organisational structure (Allchin et al., 2021). Therefore, a more comprehensive and up-to-date review would extend and refine the current knowledge base. The current review sought to identify, synthesise and appraise the qualitative literature examining adult mental health practitioners’ implementation and experiences of FFP. This review also aimed to provide an evidence-informed account of practitioners’ FFP delivery experiences and to identify key recommendations to enhance future FFP outcomes in AMHS.

## Methods

### Search Strategy and Identification of Studies

The Sample, Phenomenon of Interest, Design, Evaluation, Research Type (SPIDER) tool (Cooke et al., 2012) and the Preferred Reporting Items for Systematic Reviews and Meta-Analyses (PRISMA) guidance (Moher et al., 2009) supported the development of the search strategy. Five databases (Ovid Medline, PsycInfo, CINAHL plus, EMBASE and Web of Science Core Collection) were selected and searched up to January 2022, based on their relevance to the research aims. Search terms were informed by the titles and abstracts of key papers as well as key reviews (Foster et al., 2016; Gregg et al., 2021) to ensure comprehensive FFP-related search term inclusion. Pilot searches were undertaken to help generate the final search terms (see Table 1). Search terms were categorised into “family-focused practice”, “views and experiences”, “adult mental health practitioner” and “qualitative”. All of the categories were combined with Boolean operator “and”. Additional hand searching of identified paper’s references supported the search. The review protocol was registered with the PROSPERO international prospective register of systematic reviews (http://www.crd.york.ac.uk/prospero, registration number CRD42022306863).

**Table 1:** Search terms by category

Database (and platform)	PsycInfo (OVID); Medline (OVID), EMBACE (OVID), CINAHL plus (EBSCOhost); and Web of Science (Claravate)			
Key search categories	“Family-focused practice”	“Adult mental health practitioner”	“Views and experiences”	“Qualitative”
Search terms	OR family-centred	OR Adult psychiatric practice	OR Experience	OR Interpretative Phenomenological Analysis
	OR family driven	OR adult mental health	OR attitude*	OR IPA
	OR family focus*	OR adult mental health clinicians	OR barrier*	OR Thematic Analysis
	OR family friendly	OR clinicians	OR enabler*	OR TA
	OR family guided	OR adult mental health staff	OR facilitator	OR Grounded Theory
	OR family inclusive	OR adult psychiatry	OR experience	OR Questionnaire*
	OR family orient*	OR community mental health	OR factors	OR Survey*
	OR family sensitive	OR mental health nurses	OR views	OR Interview*
	OR family support	OR mental health professionals	OR challenges	OR Focus group*
		OR psychiatric nurses	OR understand*	OR Case stud*
		OR psychologists	OR perspectives	OR Observ*
		OR occupational therapist	OR knowledge	
		OR psychiatrist	OR practice*	
			OR thoughts*	
			OR descriptions	
			OR opinions	
			OR perceptions	
			OR enablers	

### Inclusion and Exclusion Criteria

Papers were included if: (1) participants were adult mental health practitioners who worked in AMHS, (2) studies included data on views and experiences of FFP as well as perceived barriers and facilitators, (3) studies using qualitative methods of data collection and analysis, (4) studies were written in English or German in peer-reviewed journals. Papers were excluded if: (1) participants were mental health practitioners working in child services, substance use or physical healthcare roles, or occupying a solely managerial, non-clinical position, (2) studies centred on FFP-specific interventions or projects, such as family-based interventions or any family-specific therapies (e.g., behavioural family therapy), (3) the focus of the study was not exclusively on professionals’ FFP delivery and (4) the study was a review or did not present empirical data, such as theses, opinion pieces and audits. Mixed sample studies (e.g., clients, families and practitioners) and mixed-method papers were included if practitioner data were presented independently but excluded if data were combined. Similarly, mixed-method papers were included if their qualitative data were distinct from the quantitative findings.

### Quality Appraisal

The methodological quality of the included studies was assessed by the first author using the 10-item Critical Appraisal Skills Programme (CASP) checklist for qualitative studies (available from https://casp-uk.net), a widely used quality assessment tool for assessing qualitative research (Long et al., 2020). The items on the CASP checklist were also attributed numerical outcome (see Butler et al., 2020) (No = 0, Can’t Tell = 0.5, Yes = 1), resulting in a maximum total score of 10. The total CASP score for all papers was used to categorise the methodological quality as either “high” (> 8–10), “moderate” (6–8) or “low” (≤ 5). An external rater independently appraised over 25% of the included papers, indicating substantial agreement (95.71%, kappa = 0.87), and any discrepancy was resolved through discussion.

### Thematic Synthesis

Thematic synthesis was utilised (Thomas & Harden, 2008). This method is appropriate to synthesise the findings of multiple qualitative studies (Thomas & Harden, 2008), and it has also been noted for its utility for reviews centred on barriers and facilitators to practices (Barnett-Page & Thomas, 2009). The generation of new themes allows the synthesis to extend the content of the original studies and generate further understandings (Thomas & Harden, 2008; Thorne et al., 2004).

The three stages of thematic synthesis as outlined by Thomas and Harden (2008) were undertaken by the first author and managed using NVivo software (QSR International Pty Ltd. Version 12, 2018). This included: (1) line-by-line coding of the studies’ findings, (2) the organisation of codes into related areas and (3) the development of analytical themes. All text under the headings “results” or “findings” were extracted electronically and entered into NVivo software (QSR International Pty Ltd. Version 12, 2018) where data were prepared for analysis. Study characteristics were also tabulated. The process of coding and developing descriptive and analytical themes was undertaken inductively by the first author, allowing themes to emerge organically from the data. The validity of the themes was scrutinised by the research team to minimise bias. Enhancing transparency in reporting the synthesis of qualitative research (ENTREQ) guidelines was adhered to (Tong et al., 2012).

## Results

**Fig. 1:**
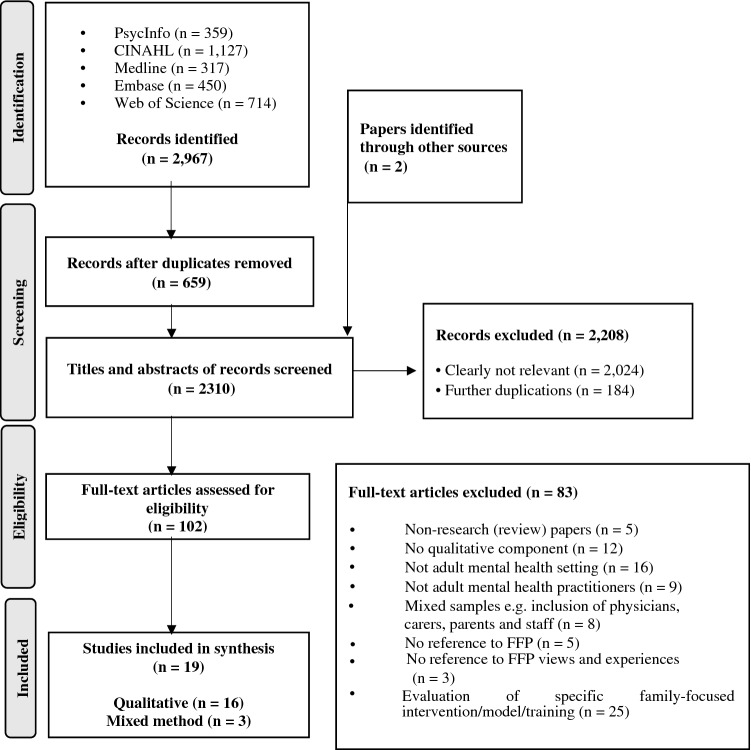
PRISMA diagram of search strategy

Figure 1 presents an outline of the search process based on PRISMA guidelines (Moher et al., 2009). The initial screening of titles and abstracts was carried out by the first author. A second reviewer, independent of the research team, screened 15% of the sample indicating substantial agreement (98.05%). At the full-text screening stage, the first author scrutinised all papers against the inclusion criteria and in cases of uncertainty, decisions regarding inclusion were made after discussion with the research team.

### Characteristics of Included Studies

A total of 19 studies were identified for inclusion in the current review as summarised in Table 2, in chronological order. Two studies were identified through forward and backward reference searching. All studies were published after 2004. Studies were conducted in Australia (*n* = 6), the UK (*n* = 3), Norway (*n* = 3), Sweden (*n* = 3), Ireland (*n* = 2), Germany (*n* = 1) or New Zealand (*n* = 1). Recruitment settings for the studies included: Community Mental Health Services (*n* = 5), Inpatient Mental Health Services (*n* = 6), Outpatient Services (*n* = 1) and mixed samples of Inpatient, Outpatient and Community Mental Health Services (*n* = 7). Samples ranged from 6 to 219 participants, with a combined sample of 496. Tchernegovski et al., (2018a, 2018b) published data from the same sample, as did Grant et al. (2019) and Grant and Reupert (2016). Therefore, the 19 studies were drawn from 17 samples.

**Table 2:** Characteristics of included studies

No.	Authors, publication year, country	Country FFP Policy	Title	Participants	Recruitment setting	Data collection	Data analysis
1	Sunde et al. (2021) Norway	Yes	Professionals’ understanding of their responsibilities in the collaboration with family caregivers of older persons with mental health problems in Norway	*n* = *18* 13 Mental Health (MH) nurses 2 Social workers 1 Child welfare 1 Occupational therapy 1 Other health and social related course	Community Mental Health Services	Focus groups	Thematic Analysis (Braun & Clark, 2006)
2	Radley et al. (2021) UK	No	Mental health professionals’ experiences of working with parents with psychosis and their families: a qualitative study	*n* = *19* 8 MH nurses 5 Social workers 2 Occupational therapists 2 Psychiatrists 1 Support worker 1 Psychotherapist	Early Intervention in Psychosis Teams & Community Mental Health Services	Focus groups	Thematic Analysis (Braun & Clark 2006)
3	Skundberg-Kletthagen et al. (2020) Norway	Yes	Mental health professionals’ experiences with applying a family-centred care focus in their clinical work	*n* = *13* 13 Mental health professionals: Nurses, Social workers, Social educators, Psychologists or Occupational Therapists	Community Mental Health Services	Semi-structured interviews	Phenomenographic Analysis (Martin et al., 1992)
4	Leenman and Arblaster (2020) Australia	Yes	Navigating rocky terrain: A thematic analysis of mental health clinician experiences of family-focused practice	*n* = *:10* 1 Occupational therapist 2 Social workers 1 Psychologist 1 Case manager/social worker 3 Team leader/registered nurses 1 Case manager/registered nurse 1 Registered nurse	Community Mental Health Services	Semi-structured interviews	Thematic Analysis (Braun & Clark 2006)
5	Krumm et al. (2019) Germany	No	Mental health nurses’ and psychiatrists’ views on addressing parenthood issues among service-users	*n* = *30* 15 Nurses 15 Psychiatrists	Inpatient Mental Health Service	Focus groups	Content Analysis (reference not provided)
6	Grant et al. (2019)* Ireland	Yes	Predictors and enablers of mental health nurses’ family-focused practice	*n* = *14* Psychiatric nurses out of 343 who completed the quantitative component	Inpatient and Community Mental Health Services	Mixed methods: Semi-structured interviews subsequent to high scores on The Family-Focused Mental Health Practice quantitative questionnaire	Thematic Analysis (Braun & Clark 2006)
7	Tchernegovski et al. (2018a) Australia	Yes	Adult mental health clinicians’ perspectives of parents with a mental illness and their children: single and dual focus approaches	*n* = *11* 4 Psychologists 2 MH nurses 3 Social workers 1 Psychiatrist 1 Occupational therapist	Inpatient Community & Outpatient Mental Health Services	Semi-structured interviews	Interpretive Phenomenological Analysis (Smith, 1996)
8	Tchernegovski et al. (2018b) Australia	Yes	How do Australian adult mental health clinicians manage the challenges of working with parental mental illness? A phenomenological study	As above (same sample as Tchernegovski et al., 2018a)	As above (same sample as Tchernegovski et al., 2018a)	Semi-structured interviews	Interpretive Phenomenological Analysis (Smith, 1996)
9	Strand and Rudolfsson (2018) Sweden	No	Professionals’ experiences of integrating a child perspective in adult psychosis service	*n* = *11* 6 Social workers 4 MH workers 1 Nurse	Outpatient Mental Health Services	Semi-structured interviews	Thematic Analysis (Braun & Clark 2006)
10	Foster and Isobel (2018) Australia	Yes	Towards relational recovery: Nurses’ practices with consumers and families with dependent children in mental health inpatient units	*n* = *20* MH nurses	Inpatient Mental Health Services	Semi-structured interviews	Thematic Analysis (Braun & Clark 2006)
11	Ward et al. (2017) Australia	Yes	Family-focused practice within a recovery framework: practitioners’ qualitative perspectives	*n* = *11* 5 MH nurses 2 Social workers 2 Social/community welfare workers 1 Psychologist 1 Occupational therapist	Inpatient and Community Mental Health Services	Semi-structured interviews	Thematic Analysis (Braun & Clark 2006)
12	Hjärthag et al. (2017) Sweden	No	Professional views of supporting relatives of mental health clients with severe mental illness	*n* = *23* Case managers, Social workers, MH nurses, Physiotherapists or Psychologists	Community Mental Health Services	Semi-structured interviews	Thematic Analysis (Braun & Clark 2006)
13	Pfeiffenberger et al. (2016)* New Zealand	Yes	The well-being of children of parents with a mental illness: the responsiveness of crisis mental health services in Wellington	*n* = *22* 1 Social worker 1 Mental health practitioner 1 Adult psychiatrist 1 Child and adolescent psychiatrist 1 Crisis assessment 1 Child protection coordinator 1 Emergency department worker 1 Paediatrician 4 Primary care 4 Government or national workers	Mental Health Crisis Services	Mixed methods: Quantitative audit and document review followed by semi-structured interviews	Thematic Analysis (Braun & Clark 2006)
14	Grant and Reupert (2016) Ireland	Yes	The impact of organizational factors and government policy on psychiatric nurses’ family-focused practice with parents who have mental illness, their dependent children, and families in Ireland	*n* = *14* MH nurses	Inpatient and Community Mental Health Services	Semi-structured interviews	Thematic Analysis (Braun & Clark 2006)
15	Lauritzen and Reedtz (2013) Norway	Yes	Support for children of service-users in Norway	*n* = *219* Staff and leaders of wards	Regional Norwegian hospital	Open-ended responses to items within a survey questionnaire (not specified)	Framework analysis (Luff & Thomas 1999)
16	O’Brien et al. (2011) Australia	Yes	Children of parents with a mental illness visiting psychiatric facilities: Perceptions of staff	*n* = *9* 3 MH nurses 2 Psychiatrists 2 Social workers 2 Occupational therapists	Inpatient Mental Health Services	Semi-structured interviews	“Qualitative exploratory research framework”
17	Maddocks et al. (2010) UK	No	A phenomenological exploration of the lived experience of mental health nurses who care for clients with enduring mental health problems who are parents	*n* = *6* MH nurses	Two inpatient long-term residential wards	Semi-structured interviews	Interpretive Phenomenological Analysis (Smith et al., 1996)
18	Slack and Webber (2008)* UK	No	Do we care? Adult mental health professionals’ attitudes towards supporting service-users’ children	*n* = *15* 15 respondents to qualitative components out of 91 in quantitative component Profession unknown	Inpatient and Community Mental Health Services	Cross-sectional survey questionnaire with Open ended responses to survey questions	Pattern coding for analysis of additional comments
19	Sjöblom et al. (2005) Sweden	No	Nurses’ view of the family in psychiatric care	*n* = *:20* MH nurses	Inpatient Services	Semi-structured interviews	Thematic Analysis (Braun & Clark 2006)

In studies marked with an * qualitative data had been collected as part of mixed-method analysis

In terms of data collection of the qualitative papers, 13 studies utilised interviews and three utilised focus groups. One study presented only the qualitative data from a survey (Lauritzen & Reedtz, 2013), having published the quantitative data in a separate paper (Lauritzen et al., 2015). Three studies utilised mixed-method designs (Grant et al., 2019; Pfeiffenberger et al., 2016; Slack & Webber, 2008). Grant et al. (2019) used the “family-focused mental health practice questionnaire” (Maybery et al., 2012) whereby participants who obtained high scores on the quantitative component were interviewed (Grant et al., 2019). Slack and Webber (2008) used a questionnaire developed for their specific research purposes which included open-ended textual responses which were analysed qualitatively. Pfeiffenberger et al. (2016) undertook an audit of the electronic clinical records, completed a documentary review and interviewed key informants. Only qualitative data were extracted from these studies. Methods of analysis included: Thematic Analysis (*n* = 11) and Interpretive Phenomenological Analysis (*n* = 3), Phenomenographic Analysis (*n* = 1), Content Analysis (*n* = 1), Framework Analysis (*n* = 1) and Pattern Coding (*n* = 1) (see Table 2). One study did not specify the method of analysis beyond stating a “qualitative exploratory research framework” was used, but it described the analytical process.

### Methodological Quality of Included Studies

The methodological quality of all included studies was appraised and deemed as high (*n* = 17) or moderate (*n* = 2). Detailed quality appraisal ratings are shown in Table 3. Overall, the studies mostly reported rigorous analysis and presented in-depth descriptions of the analytical process. A significant limitation was noted in the lack of critical acknowledgement of researcher influence in nine papers leading to potential bias (see Table 3). Notably, in two studies ethical approval was not reported. Given that there is not a widely accepted or empirically tested approach for excluding qualitative studies from synthesis on the basis of quality (Dixon-Woods et al., 2006; Thomas & Harden, 2008), no studies were excluded.

**Table 3:**
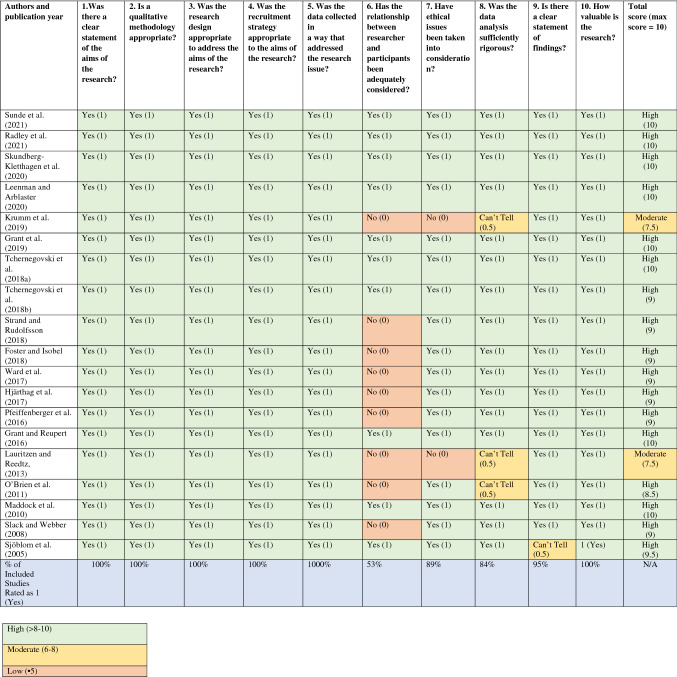
Methodological quality assessment of included studies

### Thematic Synthesis

Three main themes were developed representing different aspect of practitioners’ experience of FFP delivery: *(1) A Variable Approach, (2) A Balancing Act and (3) What Works?* Table 4 provides a detailed matrix of these three main themes and their subthemes, illustrating which themes were present in the 19 studies. *A Variable Approach* included the following subthemes: “practitioners’ beliefs about FFP” (e.g., see Beliefs about FFP column in Table 4), “practitioners’ roles and responsibilities”, “service delivery setting”, “practitioners’ competence and confidence in delivering FFP”, and “practitioners’ personal parenting experience”. *A Balancing Act* highlights the key simultaneous demands that practitioners had to balance and navigate with service-users, families and service contexts, which requires consistent negotiation and consideration throughout the delivery of FFP. Subthemes included: “the dual focus: difficulties keeping children and parents in mind”, “mutual understanding: the need to balance multiple perspectives” (e.g., see Balancing multiple perspectives column in Table 4), “navigating powerful emotions”, and “the person-centred paradox”. *What Works?* offers strategies that support practitioners FFP delivery endeavours. Subthemes included: “guidelines and regulatory frameworks”, “dedicated resources”, “a strength-based approach”, “working together” , and “inter-agency collaboration”.

**Table 4:** Matrix of included studies and identified themes

	**Theme 1: a variable approach**					**Theme 2: the balancing act**				**Theme 3: what works?**				
	**Beliefs about FFP**	**Role and responsibilities**	**Service delivery setting**	**Competence and confidence**	**Personal parenting experience**	**The dual focus**	**Balancing multiple perspectives**	**Navigating powerful emotions**	**The person-centred paradox**	**Guidance**	**Dedicated resources**	**A strength-based approach**	**Working together**	**Inter-agency collaboration**
Sunde et al. (2021)	**✓**	**✓**		**✓**		**✓**	**✓**			**✓**	**✓**	**✓**	**✓**	
Radley et al. (2021)	**✓**	**✓**	**✓**	**✓**		**✓**	**✓**	**✓**	**✓**	**✓**	**✓**	**✓**		**✓**
Skundberg-Kletthagen et al. (2020)	**✓**	**✓**				**✓**	**✓**	**✓**			**✓**	**✓**		
Leenman and Arblaster (2020)	**✓**	**✓**		**✓**	**✓**	**✓**		**✓**	**✓**		**✓**	**✓**	**✓**	
Krumm et al. (2019)	**✓**	**✓**		**✓**	**✓**			**✓**		**✓**	**✓**		**✓**	**✓**
Grant et al. (2019)	**✓**	**✓**	**✓**		**✓**				**✓**		**✓**		**✓**	
Tchernegovski et al. (2018a)	**✓**	**✓**			**✓**	**✓**		**✓**		**✓**	**✓**	**✓**	**✓**	
Tchernegovski et al. (2018b)				**✓**	**✓**	**✓**		**✓**	**✓**		**✓**	**✓**	**✓**	**✓**
Strand and Rudolfsson (2018)	**✓**	**✓**	**✓**	**✓**		**✓**		**✓**		**✓**	**✓**	**✓**		**✓**
Foster and Isobel (2018)	**✓**	**✓**	**✓**	**✓**	**✓**		**✓**	**✓**	**✓**	**✓**	**✓**			
Ward et al. (2017)	**✓**	**✓**	**✓**	**✓**			**✓**	**✓**			**✓**	**✓**		
Hjärthag et al. (2017)	**✓**	**✓**		**✓**			**✓**	**✓**			**✓**	**✓**	**✓**	**✓**
Pfeiffenberger et al. (2016)	**✓**	**✓**		**✓**		**✓**			**✓**	**✓**	**✓**		**✓**	**✓**
Grant and Reupert (2016)	**✓**	**✓**	**✓**	**✓**				**✓**	**✓**		**✓**		**✓**	**✓**
Lauritzen and Reedtz (2013)	**✓**	**✓**		**✓**		**✓**		**✓**	**✓**	**✓**	**✓**		**✓**	
O’Brien et al. (2011)	**✓**	**✓**	**✓**	**✓**			**✓**	**✓**	**✓**	**✓**	**✓**	**✓**		
Maddocks et al. (2010)	**✓**	**✓**	**✓**	**✓**		**✓**		**✓**	**✓**		**✓**			**✓**
Slack and Webber (2008)	**✓**	**✓**	**✓**	**✓**				**✓**			**✓**			**✓**
Sjöblom et al. (2005)	**✓**	**✓**	**✓**			**✓**	**✓**	**✓**			**✓**			

### Theme 1: A Variable Approach

This theme encompasses factors associated with practitioners and includes their beliefs about FFP, their perceived FFP roles and responsibilities, competence and confidence, service settings and parenting status. These factors appeared to contribute to the variability in the delivery of FFP. Five subthemes were included in the analysis.

#### Practitioner Beliefs About FFP

This subtheme illustrates that practitioner’s beliefs about FFP impacted their practice. Practitioners, in principle, acknowledged the importance of considering wider family, including children, in their service-users’ care (Grant et al., 2019; Grant & Reupert, 2016; Hjärthag et al., 2017; Leenman & Arblaster, 2020; Maddocks et al., 2010; O’Brien et al., 2011; Pfeiffenberger et al., 2016; Sjöblom et al., 2005; Skundberg-Kletthagen et al., 2020; Slack & Webber, 2008; Strand & Rudolfsson, 2018; Radley et al., 2021; Tchnernegovski et al., 2018a; Ward et al., 2017). It was described by some as a “basic attitude” (Sunde et al., 2021, p. 5) that had the ability to “break the cycle of intergenerational mental illness” (Tchernegovski et al., 2018a, p. 5) and support recovery efforts (Foster & Isobel, 2018; Sunde et al., 2021; Ward et al., 2017).

There is no one anywhere, no matter what their title or role in the organization, that doesn’t support the notion that families should be supported and that a child perspective is important: there is no one that opposes that. (Strand & Rudolfsson, 2018, p. 66)

We all know that if you don’t engage the family meaningfully, people’s recoveries are limited. (Ward et al., 2017, p. 3)

Despite reflections of FFP as important, most practitioners believed FFP was an additional duty and extension to their routine practice requiring more time and resources (Grant & Reupert, 2016; Krumm et al., 2019; Lauritzen and Reedtz, 2013; Leenman & Arblaster, 2020; Pfeiffenberger et al., 2016; Skundberg-Kletthagen et al., 2020; Strand & Rudolfsson, 2018; Sunde et al., 2021; Radley et al., 2021; Ward et al., 2017). FFP is not suitably resourced despite increased practice demands, which led to a need to prioritise elsewhere (Grant & Reupert, 2016; Lauritzen & Reedtz, 2013; Strand & Rudolfsson, 2018; Sunde et al., 2021).

Most respondents emphasised their need for adequate time to perform additional duties. (Lauritzen & Reedtz, 2013, p. 16)

Now we’re working with the child’s perspective in addition to our real job, and it just becomes an extra task like everything else. (Strand & Rudolfsson, 2018, p. 67)

It was noted that considering wider family was somewhat new practice and unfamiliar terrain (Skundberg-Kletthagen et al., 2020; Lauritzen & Reedtz, 2013; Krumm et al., 2019; Strand & Rudolfsson, 2018).

#### Practitioners’ Roles and Responsibilities

The subtheme captures how the extent to which practitioners viewed FFP as part of their role varied widely. Many practitioners supported the notion that FFP was their role and responsibility (Foster & Isobel, 2018; Grant et al., 2019; Grant & Reupert, 2016; Hjärthag et al., 2017; Leenman & Arblaster, 2020; Maddocks et al., 2010; O’Brien et al., 2011; Pfeiffenberger et al., 2016; Sjöblom et al., 2005; Skundberg-Kletthagen et al., 2020; Strand & Rudolfsson, 2018; Tchernegovski et al., 2018a; Radley et al., 2021; Ward et al., 2017). At the same time, practitioners felt opportunities to deliver FFP were “scarce” (Sunde et al., 2021, p. 3), largely due to insufficient resources (Grant et al., 2019; Krumm et al., 2019; Lauritzen and Reedtz, 2013; Skundberg-Kletthagen et al., 2020; Strand & Rudolfsson, 2018; Sunde et al., 2021).

Conversely, some participants did not believe FFP to be within their role and that working with children was outside the remit of an adult mental health practitioner (Foster & Isobel, 2018; Hjärthag et al., 2017; Maddocks et al., 2010; Radley et al., 2021; Slack & Webber, 2008). Children were described to be beyond the scope of adult mental health practitioners’ expertise and the role of child-services (Foster & Isobel, 2018; Hjärthag et al., 2017; Maddocks et al., 2010; O’Brien et al., 2011; Krumm et al., 2019; Slack & Webber, 2008; Tchernegovski et al., 2018a; Radley et al., 2021). In some studies, “impartiality” to children was advocated (Maddocks et al., 2010) and child involvement was described as “inappropriate in relation to my relationship with the parent” (Slack & Webber, 2008, p. 76). The exception to this appeared to be safeguarding, where there was ubiquitous agreement across all studies that this was the role and priority of an adult mental health practitioner.

I don’t think looking at the children is a key priority. The priority is to get the person well and recovered unless there is a clear history of abuse or serious harm to that child. (Pfeiffenberger et al., 2016, p. 604)

They’re with us with a view to help them address their symptoms and be able to recover so their primary need is to get well and obviously we expect that if we achieve that, they will be able to parent their children successfully. (Radley et al., 2021, p. 7)

Some practitioners indicated uncertainty with regard to the boundaries of their role (Foster & Isobel, 2018; Radley et al., 2021; Strand & Rudolfsson, 2018; Sunde et al., 2021) describing it as “blurry and unclear” (Strand & Rudolfsson, 2018, p. 64) and a “grey area” (Radley et al., 2021, p.7). Even studies conducted in countries where FFP was mandated, practitioners described ambiguity in relation to their roles related to FFP (Lauritzen & Reedtz, 2013; O’Brien et al., 2011; Pfeiffenberger et al., 2016; Tchernegovski et al., 2018a). Lack of role clarity meant that the provision of FFP was sporadic and inconsistently applied: “We are not good enough, or there is no system, it often becomes random when someone get support” (Sunde et al., 2021, p. 5).

#### Service Delivery Setting

This subtheme shows how the service setting might impact FFP, There seemed to be disparity as to whether service setting impacted on FFP delivery. Some practitioners in acute inpatient services adopted a “narrower focus” (Grant et al., 2019, p.147) centred on “problems” (Grant & Reupert, 2016, p. 210). Other practitioners believed the nature of inpatient environments was not “family friendly” and thus child attendance was inappropriate (Foster & Isobel, 2018; Maddocks et al., 2010; O’Brien et al., 2011; Sjöblom et al., 2005).

…they are our clients, they’re the ones in hospital and they’re the ones that we are first and foremost accountable to, so we have to put our client first. (Maddocks et al., 2010, p. 679)

There is nothing in place in acute wards that allows for the protection of these children … or to minimize the risk involved. (O’Brien et al., 2011, p. 360)

Conversely, Foster and Isobel (2018) commented on the integral nature of family inclusion during client inpatient hospitalisation through the use of family rooms: “In recovery, family is important, and visitors are important, and children, if they are important to that person, are obviously very important. So yes, it would fit in… with the philosophy of the unit” (Foster & Isobel, 2018, p. 731). Studies in Early Intervention settings by Radley et al. (2021) and Strand and Rudolfsson (2018) referred to the length of time they had with their service-users which promoted relationship development and thus facilitated FFP. Similarly, rehabilitation services were described as more suited to FFP as a result of time (Maddocks et al., 2010). Practitioners based in the community described home visits as fostering a family approach due to the insights into family functioning that they provided (Grant & Reupert, 2016) and some practitioners described community settings as having family-centred philosophies (Grant & Reupert, 2016; Grant et al., 2019), in contrast to acute hospital settings (Ward et al., 2017). However, clinic space to see families and children was also identified as a resource challenge in the community (Slack & Webber, 2008).

#### Practitioners’ Competence and Confidence in Delivering FFP

This subtheme related to practitioners’ fundamental competence and confidence in delivery FFP, and this led to variability in practice efforts. Across the studies, practitioners expressed that they lacked in competence, knowledge and confidence to deliver FFP and to meet the expectations of service-users and their wider families (Foster & Isobel, 2018; Grant & Reupert, 2016; Lauritzen & Reedtz, 2013; Krumm et al., 2019; Leenman & Arblaster, 2020; Maddocks et al., 2010; O’Brien et al., 2011; Pfeiffenberger et al., 2016; Radley et al., 2021; Slack & Webber, 2008; Sunde et al., 2021; Tchernegovski et al., 2018b; Ward et al., 2017). Practitioners made reference to their own competence being restricted to adult mental health and outside of this they felt “alienated” (Lauritzen & Reedtz, 2013, p. 15) and “out of their depth” (O’Brien et al., 2011, p. 361). Participants also commented on their limited experience of working with children (Lauritzen & Reedtz, 2013) and lack of an educational background in child-specific or family-specific work (Lauritzen & Reedtz, 2013; Leenman & Arblaster, 2020). Some practitioners noted that they were confident in identifying family members and children, but that their competence beyond this was limited (Foster & Isobel, 2018; Hjärthag et al., 2017). Training was identified as a mechanism to build FFP competence and confidence (Foster & Isobel, 2018; Grant & Reupert, 2016; Krumm et al., 2019; Lauritzen & Reedtz, 2013; Leenman & Arblaster, 2020; Maddocks et al., 2010; O’Brien et al., 2011; Pfeiffenberger et al., 2016; Radley et al., 2021; Slack & Webber, 2008; Sunde et al., 2021; Tchernegovski et al., 2018b; Ward et al., 2017).

Furthermore, participants who had undergone training in formal family interventions also commented on their lack of confidence to deliver these interventions in “real-world clinical practice” (Hjärthag et al., 2017). Clinical experience was described as guiding practice in one study (Leenman and Arblaster, 2020). Knowledge gaps in the role of other services and referral pathways were also identified as a training need (Grant & Reupert, 2016; Strand & Rudolfsson, 2018).

I’m not confident at it because I am not trained in it. I’m not qualified to give family-centred care. (Maddocks et al., 2010, p. 5)

We need information about building competency to manage complex family and parenting issues, how to put the family at the centre of care and recovery and how to refer to family services and how to utilize voluntary sector support. (Grant & Reupert, 2016, p. 211)

#### Practitioners’ Personal Parenting Experience

How practitioners’ parenting status impacted their delivery of FFP was outlined within this subtheme. Practitioners identified that their personal experience of caring for their own children increased their awareness, skills and knowledge of service-users’ needs as parents and the needs of a child (Foster & Isobel, 2018; Grant et al., 2019; Krumm et al., 2019; Leenman & Arblaster, 2020; Tchernegovski et al., 2018a, 2018b): “My own experience as a parent gives me the insight into how challenging parenting can be and without that insight I would find it difficult” (Grant et al., 2019, p. 145). Studies also commented that this experience increased practitioner confidence (Foster & Isobel, 2018) and supported the practical approach they took: “I talk to children in the way I talk to my own children” (Grant et al., 2019, p.145) as well as using their personal experience as a way to empathise and share experiences (Leenman & Arblaster, 2020). Participants who were not parents explicitly described FFP as difficult: “I don’t have children myself so it can be difficult… to understand the challenges of parents or to advise parents” (Grant et al., 2019, p. 145). Those who were not parents drew on years of professional experience (Leenman & Arblaster, 2020).

Despite the recognition of the value of FFP across most studies, a lack of role clarity and clear responsibilities, coupled with feelings of incompetence and insufficient resources, meant that FFP varied considerably across practitioners. The diversity of practitioners’ personal parenting experience also contributed to this variability.

### Theme 2: A Balancing Act

This theme highlights the interrelationships of practitioners with service-users, families and services, resulting in an ongoing balancing act in the delivery of FFP. The need to hold service-users and children in mind was expressed as a core challenge. The powerful feelings that FFP evoked in practitioners in combination with the emotions of service-users required balance, as did the key tension between the “person-centred” model of AMHS delivery and FFP.

#### The Dual Focus: Difficulties Keeping Both Parents and Children in Mind

This subtheme outlines practitioners’ experiences of having to balance and hold in mind both parents and children. Keeping both parents and children in mind was a key challenge for practitioners, particularly balancing alliance with the parent service-user with the needs of children (Leenman & Arblaster, 2020; Maddocks et al., 2010; Sjöblom et al., 2005; Strand & Rudolfsson, 2018; Sunde et al., 2021; Tchernegovski et al., 2018a, 2018b). It was recognised that this dual focus was necessary (Skundberg-Kletthagen et al., 2020): “there needs to be a focus on the child outcomes and wellbeing and there needs to be a focus on the parent outcomes and wellbeing” (Tchernegovski et al., 2018a, p. 5), but the process could feel conflicting and like a “rocky terrain” (Leenman & Arblaster, 2020, p. 75). This dual focus was variously described as “a real balancing act”, “walking a line”, a “tight rope” and a “push and pull” (Tchernegovski et al., 2018a, p. 5), particularly when risk was involved and actions could have relationship consequences with the service-user (Sunde et al., 2021). The absence of a balancing act and a disproportionate “single focus” (Tchernegovski et al., 2018a, p. 5) on either the child or the parent had the propensity for detrimental consequences (Tchernegovski et al., 2018a).

Some practitioners described an “impartiality”(Maddocks et al., 2010, p. 678) to children and a “parents focus” (Tchernegovski et al., 2018a, p. 5) as their role (Maddocks et al., 2010; Pfeiffenberger et al., 2016; Radley et al., 2021; Tchernegovski et al., 2018a, 2018b), but there was recognition of this alliance-making practitioners “blind to shortcomings” (Strand & Rudolfsson, 2018, p. 64) and being a challenge when having to raise risk issues due to fears of betraying service-users: “ you get a call from child protection and all of a sudden you just want to put a ‘shut‐up shop’” (Tchernegovski et al., 2018b, p. 385). Similarly, other practitioners described a singular focus on children triggered by sympathetic feelings (Tchernegovski et al., 2018a), which was also deemed as inadequate. Practitioners’ attempts to balance both parents and children were hindered by organisational models of care and insufficient resources that fostered an “individualised treatment approach” (Lauritzen & Reedtz, 2013, p. 76).

You’re trying to find that balance all the time between acting safely and not overly sort of escalating things because even if it’s intended to be protective, it can really increase anxiety levels. (Radley et al., 2021, p. 7)

I used to find it quite difficult because you are with your client and you want to support them but you have to think about the child, you have to think about their safety, their future and their emotional needs as well.... (Maddocks et al., 2010, p. 677)

#### Mutual Understanding: The Need to Balance Multiple Perspectives

The subtheme captures how FFP requires practitioners to draw together and balance a range of perspectives from service-users and family members, which are not necessarily harmonised or consistent. A key task of a practitioner is to reflect and balance these multiple perspectives in their actions and care planning. This can be a challenge and a “dilemma” (Sunde et al., 2021, p. 6), when there are conflicting views regarding aetiology and prognosis of service-users’ distress. Conflict was not always inevitable; however, practitioners readily identified the value of information sharing with families through psychoeducation in order to support the development of a “mutual understanding” and reduce feelings of stigma (Foster & Isobel, 2018; Hjärthag et al., 2017; O’Brien et al., 2011; Skundberg-Kletthagen et al., 2020). This process supported service-user recovery efforts by reducing family expectations of service-users (Skundberg-Kletthagen et al., 2020), improving family insight and compassion (Sunde et al., 2021) and enhancing family communication (Ward et al., 2017). Ultimately, it also facilitated family engagement in care. Reciprocally, practitioners identified the benefit of information from family members; both background information given their historical knowledge, as well as “real-time” observations (O’Brien et al., 2011; Radley et al., 2021; Sjöblom et al., 2005; Skundberg-Kletthagen et al., 2020; Sunde et al., 2021; Ward et al., 2017).

Family caregivers have a lot of knowledge, through a long life, which can be important to us… they are a great resource, they are in the same house and are present round the clock, and they can observe changes. (Sunde et al., 2021, p. 4)

I also think that relatives greatly need to get an explanation as to what happened and why and be able to reduce the shame and guilt. (Hjärthag et al., 2017, p. 65)

#### Navigating Powerful Emotions

The presence of heightened emotion from all stakeholders involved in the delivery of FFP defined this subtheme. Practitioners described the emotional cost of FFP when working with parents and children (Grant & Reupert, 2016; Maddocks et al., 2010; O’Brien et al., 2011; Tchernegovski et al., 2018a, 2018b; Sjöblom et al., 2005; Strand & Rudolfsson, 2018). Empathy was emotionally taxing (Grant & Reupert, 2016) and practitioners described “disappointment and anger because the care system is not able to do more” (Sjöblom et al., 2005, p. 565), despite their commitment. Practitioners described feeling “devastated”, “frustrated,” and “guilty” (Strand & Rudolfsson, 2018, p. 70) at their inability to “do more”. Practitioners also referred to their fears of damaging their relationships with service-users through the perceived “sensitive” nature of family work (Lauritzen & Reedtz, 2013; Maddocks et al., 2010; Strand & Rudolfsson, 2018; Tchernegovski et al., 2018a, 2018b).

You can’t work with parents unless you’ve an emotional connection with them and there’s a downside to having it [emotional connection]. I don’t think that’s appreciated and it would be better for us as professionals if it was acknowledged by the organization. (Grant & Reupert, 2016, p. 211)

It’s so hard and frustrating. I had a mother of five children where I made home visits, so I met the kids because they were also at home. One day I had to be there when the police took her and her new-born baby because she was totally insane, so terrible . . . I just went home and cried for days [cries]. (Strand & Rudolfsson, 2018, p. 70)

I don’t have the time, I constantly feel guilty because I don’t have the time for it, and yes it feels terrible. (Strand & Rudolfsson, 2018, p. 70).

It gives you a few sleepless nights because you wonder what the impact will be on the parents. (Tchernegovski et al., 2018a, p. 7)

Practitioners had to balance their own emotional responses with the emotions of their service-users. In particular, service-users’ expressions of guilt and shame in relation to their parenting responsibilities (Leenman & Arblaster, 2020; Maddocks et al., 2010; Radley et al., 2021; Skundberg-Kletthagen et al., 2020; Strand & Rudolfsson, 2018; Tchnernegovski et al., 2018b) and fear in relation to the implication of service involvement with their children (Krumm et al., 2019; Lauritzen & Reedtz, 2013; Leenman & Arblaster, 2020; Strand & Rudolfsson, 2018; Tchernegovski et al., 2018b; Ward et al., 2017). This shame and fear can lead to FFP resistance as service-users find it difficult to talk about and can be “quite guarded” (Tchernegovski et al., 2018b, p. 384). Reciprocally, practitioners can be inclined to avoid a “really sensitive and an emotionally laden topic” (Tchernegovski et al., 2018b, p. 384), given the potential for disengagement and damage to the therapeutic alliance (Lauritzen & Reedtz, 2013; Maddocks et al., 2010; Slack & Webber, 2008; Strand & Rudolfsson, 2018; Tchernegovski et al., 2018b).

I know I certainly can think of a number of parents who feel incredibly guilty and actually largely this guilt is of not being able to do the parenting role as well as they would like to. (Radley et al., 2021, p. 4)

Family members were described as experiencing shame, guilt and stigmatisation (Foster & Isobel, 2018; Hjärthag et al., 2017; Sjöblom et al., 2005; Strand & Rudolfsson, 2018; Ward et al., 2017), which created family conflict and impacted engagement with FFP negatively (Skundberg-Kletthagen et al., 2020). This observation by participants was compounded by their clients’ experiences of service mistrust (Hjärthag et al., 2017; Radley et al., 2021; Sjöblom et al., 2005).

They are afraid of being accused of not being a good parent, and then be the cause of the son or daughter’s mental health problems. (Skundberg-Kletthagen et al., 2020, p. 819)

Practitioners navigated a plethora of emotions when delivering FFP and had to be attuned to their own emotional experiences and that of others which was described as a “balancing art” (Skundberg-Kletthagen et al., 2020, p. 819).

#### The Person-Centred Paradox

Conventionally, AMHS have adopted a “problem-focused” (Grant & Reupert, 2016, p. 210) biomedical model of care, which was centred on an individual: “the mental health system is very much about your diagnosis and medication. That sort of holistic picture of a person can get missed out…” (Tchernegovski et al., 2018b, p. 384). A “person-centred” ethos has been actively promoted and for good reason in AMHS; however, the narrative of “person-centred care over family-centred care…the focus is on the patient and their illness…” (Maddocks et al., 2010, p. 678) suggested that this ethos could inhibit FFP. FFP is fundamentally counterintuitive against this background given its effectiveness relies on the involvement of multiple participants in the context of the service-users’ life. Participants commonly reported a demand for individualised treatment within their work (Foster & Isobel, 2018; Grant & Reupert, 2016; Grant et al., 2019; Lauritzen & Reedtz, 2013; Leenman & Arblaster, 2020; Maddocks et al., 2010; Radley et al., 2021; Tchernegovski et al., 2018b). Consequently, FFP could be overlooked and not prioritised: “it’s very much focused on the individual and how they manage and how they treat their presenting illness and I think at times families do get forgotten” (Leenman & Arblaster, 2020, p. 76). AMHS funding protocols were also reflected on, particularly that they provided resources for the treatment of “one index patient” (Pfeiffenberger et al., 2016, p. 603). Practitioners therefore described increased expectations with an absence of additional structural resources (Lauritzen & Reedtz, 2013; Leenman & Arblaster, 2020; Pfeiffenberger et al., 2016; Radley et al., 2021). This service expectation of ‘doing more with less’ required practitioners to balance service scope against resource constraints.

If funding went to a family rather than just the individual, that would be one of the biggest changes in the current system. (Pfeiffenberger et al., 2016, p. 603)

### Theme 3: What Works?

This theme brings together the potentially modifiable factors that could encourage adoption of and facilitate the delivery of FFP and foster a service ethos vested in FFP.

#### Guidance and Regulatory Frameworks

This subtheme is characterised by the impact of guidance and regulatory frameworks on the delivery of FFP. Practitioners reflected that there was a lack of FFP guidance or an unawareness of guidance in countries where FFP was a part of service policy (Foster & Isobel, 2018; Lauritzen & Reedtz, 2013; O’Brien et al., 2011; Pfeiffenberger et al., 2016; Strand & Rudolfsson, 2018; Tchernegovski et al., 2018a). The absence of formalised guidance and an associated regulatory framework led practitioners to feel vulnerable to operational risks in the delivery of FFP, given their lack of perceived knowledge and training, which in turn could lead to reticence in FFP application and thus compromised delivery (Maddocks et al., 2010; O’Brien et al., 2011; Radley et al., 2021; Sunde et al., 2021). There was a demand for more formalised guidance and supportive systems, particularly for recording the identification of and contact with children (Lauritzen & Reedtz, 2013; O'Brien et al., 2011; Pfeiffenberger et al., 2016). Formal FFP guidance and supportive systems would reduce arbitrary approaches to FFP (Krumm et al., 2019). On the other hand, some practitioners reflected on the burden of guidelines and that an overly structured framework might inhibit innovation and responsiveness to service-user needs: “Please, please no. Because guidelines would mean that I could be prosecuted” (Krumm et al., 2019, p. 431).

#### Dedicated Resources

The subtheme captures the need for dedicated resources in order to effectively deliver FFP. Additional “concrete resources” (Grant & Reupert, 2016, p. 208) were a pivotal prerequisite to delivery of FFP across all studies. One recommended resource was a devoted team member who was FFP-trained and could promote, educate, support and deliver FFP (Lauritzen & Reedtz, 2013; Leenman & Arblaster, 2020; O’Brien et al., 2011: Pfeiffenberger et al., 2016; Strand & Rudolfsson, 2018; Tchernegovski et al., 2018a). A “very stable and active presence” of dedicated staff had been evidenced to improve family inclusion (Leenman & Arblaster, 2020, p. 78). Furthermore, dedicated FFP staff appeared to demonstrate service commitment to FFP and fostered a unified approach (Leenman & Arblaster, 2020). One study commented that when staff who were responsible for FFP had left the service, they were not replaced which was perceived to signal that it was not a priority (Strand & Rudolfsson, 2018).

It was easier when the child’s perspective developers were here and you could receive guidance and raise questions, it was a natural source of help. Although I’m not completely alone now, there’s no one to seek guidance from, no one to lean on in difficult decisions. (Strand & Rudolffson, 2018, p. 67)

#### A Strength-Based Approach

The subtheme related to how the engagement approach that practitioners’ adopted impacted service-users’ and families’ responsiveness to FFP. Practitioners noted that seeing strengths and focusing on “potential and possibility” (Tchernegovski et al., 2018a, p. 5) facilitated service-user and family engagement (Sunde et al., 2021; Ward et al., 2017). This approach allowed practitioners to “see more love than I expected” (Skundberg-Kletthagen et al., 2020, p. 818). It also helped practitioners hold on to hope (Ward et al., 2017): “… using the strengths-based words and reminding them of the hope that there is … and I think working towards those goals step-by-step also helps clinicians to remind themselves that there is hope and that it’s not just an endless cycle” (Tchernegovski et al., 2018a, p. 6). Practitioners also referred to the need to build trust and an alliance as a fundamental prerequisite to FFP (Hjärthag et al., 2017; Leenman & Arblaster, 2020; Maddocks et al., 2010; Strand & Rudolffson, 2018; Tchernegovski et al., 2018b). Strategies for this appeared to be transparency and collaboration which allowed service-users to understand service expectations (e.g., in relation to safeguarding) and to have control and choice (Hjärthag et al., 2017; Maddocks et al., 2010; Radley et al., 2021; Skundberg-Kletthagen et al., 2020; Tchernegovski et al., 2018b; Ward et al., 2017). This approach had the potential to counteract service mistrust (Maddocks et al., 2010; Radley et al., 2021).

#### Working Together

This subtheme underscores the importance of staff teams working collaboratively to deliver FFP. Working together was seen to enhance FFP and facilitate “supportive and unified teams” (Leenman & Arblaster, 2020, p. 78). Practitioners reflected on the value of perceived support from their colleagues and management to provide guidance, direction and emotional support (Grant & Reupert, 2016; Grant et al., 2019; Leenman & Arblaster, 2020; Tchernegovski et al., 2018a, 2018b) particularly in times of challenge: “when we have got a family we are working with and finding it difficult we will seek out each other” (Leenman & Arblaster, 2020, p. 78). Multidisciplinary team structures were an enabler of FFP due to the bringing together of multiple perspectives and skills from a variety of disciplines (Grant & Reupert, 2016). Formal mechanisms for support that enhanced FFP included individual supervision, team supervision and multidisciplinary team meetings, which provided platforms for discussion, objective view taking, validation and reassurance (Hjärthag et al., 2017; Krumm et al., 2019; Strand & Rudolsson, 2018; Sunde et al., 2021; Tchernegovski et al., 2018a, 2018b). Studies commented that a lack of commitment from managers and leaders has a “domino effect” (Lauritzen & Reedtz, 2013, p. 15), evidencing the importance of vested leadership that role models a commitment to FFP (Grant & Reupert, 2016; Lauritzen & Reedtz, 2013; Pfeiffenberger et al., 2016; Tchernegovski et al., 2018a, 2018b).

#### Inter-Agency Collaboration

The role of inter-agency working in order to deliver FFP and improve outcomes for service-users and families was highlighted in this subtheme. Practitioners identified a need for enhanced cooperation and collaboration with relevant external agencies to improve service integration (Grant & Reupert, 2016; Krumm et al., 2019; Maddocks et al., 2010). In particular, there seemed to be a need for improved links with child mental health services and social services (Maddocks et al., 2010; Pfeiffenberger et al., 2016), “we need closer working relationships with social services or knowing the process” (Maddocks et al., 2010, p. 679). Studies regularly cited that practitioners refer children on to other services: “We do nothing for these kids. We just wait till they get over it themselves or till they get bad enough to refer them onto another service” (Pfeiffenberger et al., 2016, p. 603), but there seemed to be a lack of awareness of what support services were available (Krumm et al., 2019; Maddocks et al., 2010; Pfeiffenberger et al., 2016). Of those that they were aware of, their responsiveness was described as often inadequate and unpredictable (Radley et al., 2021; Strand & Rudolfsson, 2018; Tchernegovski et al., 2018b), “I do refer the children and families… but do not always get the support I expected” (Slack & Webber, 2008, p. 76). An increased willingness from all agencies, an awareness and understanding of service roles, and structures/pathways between services to support collaboration were noted to be crucial to enhance FFP delivery (Hjärthag et al., 2017; Krumm et al., 2019; Maddocks et al., 2010; Pfeiffenberger et al., 2016; Radley et al., 2021; Tchernegovski et al., 2018b).

## Discussion

This systematic literature review of 19 studies based on 17 samples provides a comprehensive synthesis of the experience of adult mental practitioners in delivering FFP. The aims of the review were fully met, and our findings resulted in the identification of key themes. Practitioners’ approach to FFP was reported to be “variable” and influenced by their beliefs in FFP, perceived FFP roles and responsibilities, competence in FFP delivery, service setting, and personal parenting status. Practitioners had to engage in a “balancing act” to navigate powerful emotions, maintain a “dual focus” on parents and children, whilst considering multiple perspectives in an organisational structure that advocates biomedical individualised treatment approaches. The studies helped to identify “what works” to enhance FFP. Although working together supported unified teams internally, a need for interagency collaboration development was identified. The use of strength-based approaches with clients and dedicated staff resource, within clear guidelines and frameworks, was necessary to maximise FFP delivery. Whilst corroborating and extending the findings of Gregg et al. (2021), Shah-Anwar et al. (2019) and Allchin et al. (2021), this metasynthesis also provides a novel “balancing” conceptualisation of the navigation between practitioners, service-users and their families, and organisational contexts.

Beliefs about FFP and perceptions of roles and responsibilities influenced FFP delivery. This finding supports previous research findings (Gregg et al., 2021; Maybery & Reupert, 2009; Maybery et al., 2016; Shah-Anwar et al., 2019); it is unsurprising given that positive attitudes and role clarity have also been found to increase the willingness of practitioners’ delivery of FFP (Foster et al., 2016; Maddocks et al., 2010; Reupert et al., 2021). Even when practitioners valued FFP and considered it their role, inadequate resources led to the need to prioritise and therefore FFP was compromised (Gregg et al., 2021; Maybery & Reupert, 2009; Maybery et al., 2016; Shah-Anwar et al., 2019).

The impact of practitioners’ perceived competence and confidence on the delivery of FFP has been consistently demonstrated in the previous research (Grant et al., 2019; Gregg et al., 2021; Leonard et al., 2018; Maybery & Reupert, 2009; Maybery et al., 2016; Shah-Anwar et al., 2019). Practitioners’ perceptions of themselves as skilled and knowledgeable has been linked to the increased use of family-focused approaches (Goodyear et al., 2017; Gregg et al., 2021; Maybery et al., 2016). Studies in this review consistently identified “knowledge-practice” gaps in relation to FFP which impeded FFP delivery. Consistent with other research, practitioners described training as a mechanism to build competence (Allchin et al., 2021; Gregg et al., 2021; Maybery & Reupert, 2009; Maybery et al., 2016; Reupert et al., 2021). The importance of training having “real-world” skill-based application is an important finding and one which corroborates Maybery et al.’s findings (2016). Future research should examine the specifics of professionals’ FFP knowledge gaps and invest in FFP training initiatives.

Comparably to Gregg et al. (2021), there did not appear to be any consensus as to whether service setting impacted FFP delivery. However, aspects of services were noted to foster FFP; for example, the provision of home visits in community mental health settings (Grant et al., 2019; Leonard et al., 2018; Shah-Anwar et al., 2019). Further research should focus on the role of service setting more extensively to ensure FFP provisions are tailored to unique service structures (Skogøy et al., 2018). In addition, the parenting status of practitioners was associated with increased engagement with FFP and thus deliver in many of the studies. Although this has been acknowledged in the literature (Grant et al., 2019; Leenman & Arblaster, 2020), no previous review has identified this as an important personal practitioner characteristic. Practitioners without children reported drawing on professional experience which was highlighted as an influential factor in FFP by Gregg et al. (2021).

This current review highlights the interrelationship of practitioners with other FFP stakeholders. Although there has been recognition for the significant role of the “dual focus” and “seeing double” (Allchin et al., 2021; Cousins, 2004; Fleck-Henderson, 2000), the implications of multiple participants (Allchin et al., 2021; Shah-Anwar et al., 2019), the emotional costs of FFP, and the biomedical, individualistic organisational models that FFP is often delivered in (Allchin et al., 2021), this review postulates a more complex dynamic: practitioners are involved in a constant interaction with FFP stakeholders in which practitioners need to perform an ongoing negotiation and “balancing act” to achieve meaningful FFP outcomes.

This finding contradicts the linear proposition presented by Maybery and Reupert (2009). The linear proposition reflects the progression from organisational policies through to clinical practice and service delivery to the client. Maybery and Reupert (2009) express this as a hierarchy whereby successive activity is dependent on the implementation of lower factors. The linear approach underplays the real challenges experienced by practitioners day-to-day in trying to meet service expectations and support families in need. The current review emphasises the need to address the multiplicity of FFP drivers rather than prioritising any factor in isolation. For example, a simple increased level of service resources (whilst necessary) would not be sufficient to support a vested FFP service ethos. The more recent findings by Maybery et al. (2016) and Gregg et al. (2021) support this contradiction of Maybery and Reupert (2009), and they also provide evidence for a dynamic inter-relationship between factors associated with practitioners, families and service-users, and wider workplace systems, rather than as a linear, hierarchical process. The intersectionality of these factors is supported by the sustainability model developed by Allchin et al. (2021), which also supports the notion that an isolated view of actions is inadequate to enhance FFP because sustainability relies on the interaction of multiple systems. Although a growing body of evidence supports this conceptualisation, but more research to examine this interconnection is required.

### Clinical Implications and Recommendations

Several facilitators of FFP were identified by practitioners. Whilst lack of resources was ubiquitous (Maybery & Reupert, 2009), this review highlighted the value of dedicated staff resource for FFP (Maybery et al., 2016). Dedicated staff supports practical FFP delivery but also demonstrates service FFP commitment (Reupert et al., 2021). Interagency collaboration was also identified as a development opportunity in this review, and an absence of liaison between services was reflected as a significant barrier to FFP (Maybery & Reupert, 2009; Maybery et al., 2016; Shah-Anwar et al., 2019). Improved awareness of other service provisions and roles as well as interagency structures to support integrated care is recommended (Reupert & Maybery, 2008). The benefits of multidisciplinary team structures, team support and management vested in FFP corroborated previous reviews (Gregg et al., 2021; Maybery & Reupert, 2009; Maybery et al., 2016). Formal workplace structures such as multidisciplinary team meetings and supervision facilitated FFP due to the supportive and development function they provided. Time should be ring-fenced to protect and prioritise these forums to build FFP capacity. A prominent theme, in line with the previous research, was the call for formal guidance and frameworks that set out practitioner FFP role expectations (see Reupert et al., 2021). Furthermore, this guidance needs to include supporting operational systems for FFP delivery (Allchin et al., 2021; Gregg et al., 2021; Leonard et al., 2018; Shah-Anwar et al., 2019). This should be a key focus for future research, service policy and practice development internationally.

Although the recommendations in this study are necessary and provide a starting point for FFP, they are not sufficient in isolation. In line with findings by Eassom et al. (2014) and Allchin et al. (2021), this review advocates for a “whole-team”, “whole-organisation” approach as the way forward to maximise FFP, mirroring the “whole-family approach” that we should be adopting with service-users and their families (Foster et al., 2016).

### Strengths and Weaknesses

Given that the search was limited to studies written in English or German and those published in peer-reviewed journals, publication and language biases are acknowledged. The language selection was based on expertise within the research team and due to a lack of funding for translation services. However, the search did not identify any non-English papers. In addition, a variety of studies from different countries and cultures were identified and included, albeit largely countries from the anglosphere, which raises the question as to how FFP is interpreted and delivered cross-culturally (Sin et al., 2017; Tungpunkom et al., 2017; Yao et al., 2021). Given the emerging international emphasis on FFP, it is important to consider the broader international landscape and further research into the delivery of FFP in specific cultures would add to the body of literature (Grant et al., 2016). Ironically, this is also a relative strength of the current review. The number and quality of the included studies provided a comprehensive exploration of adult mental health practitioners’ experiences of FFP. Although large sample sizes have been noted to impede the depth of a metasynthesis (Sandelowski et al., 1997), the use of NVivo software facilitated the systematic analysis of large amounts of data and independent review at stages of study selection, quality assessment and theme identification enhanced methodological rigour (Tong et al., 2012). Although the researchers’ roles in the study were objective, qualitative data analysis is interpretative (Denzin & Lincoln., 2003). Consequently, the validity of the results can be impacted by researcher biases (Costa et al., 2016; Fink, 2000). All researchers were white women with professional experience and training in psychology applied to research, academia and/or clinical roles. Notably, two of the researchers are parents themselves, and all authors had a vested interest and positive view of FFP. Supervision ensured the synthesis process was as transparent and reflective as possible through reflective discussions and journals. Similarly, this reflexivity encouraged an attentiveness to the diversity of epistemologies and qualitative methods employed across the studies and their interpretive contribution.

## Conclusions

This review is the largest and most comprehensive review of the qualitative literature pertaining to adult mental health practitioners’ experiences of FFP to date. The findings provided an evidence-informed account of factors that influence their practice. We demonstrated that practitioners were involved in a complex and persistent navigation between FFP stakeholders in which required a ‘balancing act’ to achieve meaningful FFP outcomes. In the light of the findings, important considerations for service development are highlighted to improve implementation of FFP in AMHS to support practitioners in this “balancing act”. Key recommendations include the implementation of policy to set out roles and practitioners’ expectations of FFP, the provision of “real-world” FFP training, the development and protection of team-working forums, the provision of dedicated FFP staff and the development of interagency awareness and pathways for integrated care structures. Overall, a “whole-team”, “whole-service” approach to FFP is advocated by this review to signify that “family matters” and to lead to long-lasting changes for our service-users and their families.
